# Association of six YFP-myosin XI-tail fusions with mobile plant cell organelles

**DOI:** 10.1186/1471-2229-7-6

**Published:** 2007-02-09

**Authors:** Daniel Reisen, Maureen R Hanson

**Affiliations:** 1 Department of Molecular Biology and Genetics, 321 Biotechnology Building, Cornell University, Ithaca, NY 14853, USA; 2 Bitplane AG, Badenerstrasse 682, CH-8048, Zurich, Switzerland

## Abstract

**Background:**

Myosins are molecular motors that carry cargo on actin filaments in eukaryotic cells. Seventeen myosin genes have been identified in the nuclear genome of *Arabidopsis*. The myosin genes can be divided into two plant-specific subfamilies, class VIII with four members and class XI with 13 members. Class XI myosins are related to animal and fungal myosin class V that are responsible for movement of particular vesicles and organelles. Organelle localization of only one of the 13 *Arabidopsis *myosin XI (myosin XI-6; At MYA2), which is found on peroxisomes, has so far been reported. Little information is available concerning the remaining 12 class XI myosins.

**Results:**

We investigated 6 of the 13 class XI *Arabidopsis *myosins. cDNAs corresponding to the tail region of 6 myosin genes were generated and incorporated into a vector to encode YFP-myosin tail fusion proteins lacking the motor domain. Chimeric genes incorporating tail regions of myosin XI-5 (At MYA1), myosin XI-6 (At MYA2), myosin XI-8 (At XI-B), myosin XI-15 (At XI-I), myosin XI-16 (At XI-J) and myosin XI-17 (At XI-K) were expressed transiently. All YFP-myosin-tail fusion proteins were targeted to small organelles ranging in size from 0.5 to 3.0 μm. Despite the absence of a motor domain, the fluorescently-labeled organelles were motile in most cells. Tail cropping experiments demonstrated that the coiled-coil region was required for specific localization and shorter tail regions were inadequate for targeting. Myosin XI-6 (At MYA2), previously reported to localize to peroxisomes by immunofluorescence, labeled both peroxisomes and vesicles when expressed as a YFP-tail fusion. None of the 6 YFP-myosin tail fusions interacted with chloroplasts, and only one YFP-tail fusion appeared to sometimes co-localize with fluorescent proteins targeted to Golgi and mitochondria.

**Conclusion:**

6 myosin XI tails, extending from the coiled-coil region to the C-terminus, label specific vesicles and/or organelles when transiently expressed as YFP fusions in plant cells. Although comparable constructs lacking the motor domain result in a dominant negative effect on organelle motility in animal systems, the plant organelles remained motile. YFP-myosin tail fusions provide specific labeling for vesicles of unknown composition, whose identity can be investigated in future studies.

## Background

Intracellular motility of organelles and transport vesicles is critical for optimization of photosynthesis and metabolism. The dynamic nature of mitochondria [1,2], chloroplasts [3], non-green plastids [4], peroxisomes [5,6], and Golgi bodies [7] has been documented through chlorophyll or fluorescent protein labeling of the organelles. Though inhibitor studies [5,6,8-10] indicate that the actin cytoskeleton is important for motility of all of these organelles, little information is available on the motor proteins responsible for movement of particular cargoes in plants.

Myosins are molecular motors carrying cargoes on actin filaments in eukaryotic cells [11-13]. Myosins have three common domains: a highly conserved motor domain located at the N-terminus which interacts with actin and hydrolyses ATP; an IQ domain which binds calmodulin or calmodulin-related proteins; a tail which varies by length and structure and which contains a coiled-coil domain consisting of alpha-helices for protein dimerization [14]. When the *Arabidopsis *genome sequence became available, a total of 17 myosin-like genes were identified [15-17]. They fall into 2 classes: myosin class VIII containing 4 genes and myosin class XI containing 13 members [15]. In the complete rice genome sequence, 2 class VI and 12 class XI myosins were detected [18]. Class VIII myosins were predicted to be involved in new cell wall formation and transport in the plasmodesmata [19], while class XI myosins, which are closely related to animal and fungal myosin class V [20], were considered likely to be involved in vesicle and organelle movement.

There may actually be more than 13 myosin XIs present in the *Arabidopsis *cell, as myosin genes are quite large, with many exons and introns that might undergo alternative splicing. In animals, alternative splicing allows the same gene to encode different myosins that have different cargo-binding capabilities [21]. In plants, myosin transcript data is still quite limited even in plant systems with abundant genomic resources. That alternative splicing does occur in plant myosin transcripts has recently been shown by the sequencing of two cDNAs corresponding to alternatively spliced transcripts of a single rice myosin XI gene [18].

In order to investigate whether members of the myosin XI gene family in plants localize to specific cargoes, we made expression constructs in which the motor domain of the myosin was replaced by yellow fluorescent protein (YFP). We have examined the localization of 6 different YFP-myosin tail fusions expressed transiently, each encoded by a different myosin XI gene. We have determined how much of the tail region is required for specific labeling of organelles and have evaluated the motility of the labeled organelles. We have investigated whether any organelles labeled with the YFP-tails co-localize with mitochondria, plastids, peroxisomes, or Golgi.

## Results and discussion

### Fluorescent protein markers for transient expression

**Figure 1:**
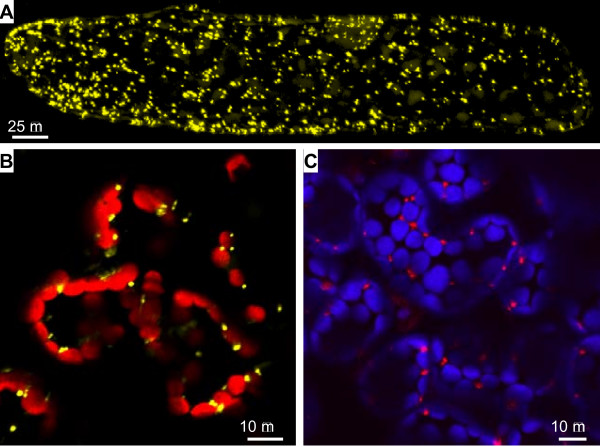
**YFP::catalase and DsRed2::catalase label peroxisomes**. **A) **Transient expression of *YFP::catalase *(yellow) in onion cells 24 h after bombardment. Maximum projection (cell depth = 92 μm) of 50 z-stack series. **B) **Transient expression of *YFP::catalase *(yellow) in tobacco leaves 48 h after *Agrobacterium *infiltration. Autofluorescence of chloroplasts is pseudo-colored in red. **C) **Transient expression of *DsRed2::catalase *(red) in tobacco leaves 48 h after *Agrobacterium *infiltration. Autofluorescence of chloroplasts is pseudo-colored in blue.

In order to examine possible localization of the YFP-tail fusions to mitochondria, peroxisomes, and Golgi bodies, we needed constructs expressing fluorescent proteins known to localize to these compartments. Previously we had shown that a yeast coxIV transit sequence fused to GFP resulted in specific labeling of mitochondria [2]. For specific labeling of Golgi bodies, we obtained the *ERD2::GFP *construct that Boevink *et al *[22] used to describe the remarkable motility of plant Golgi stacks. In order to label perixosomes, we decided to make fusions of a peroxisomal resident enzyme, catalase, which has the uncleavable tripeptide of the PTS1 (Peroxisomal Targeting Signal 1) at the C-terminus [23,24]. We produced N-terminal YFP or DsRed2 fusion genes with At catalase 2. After transient expression, both *YFP::catalase2 *and *DsRed2::catalase2 *constructs did label peroxisomes in onion cells (Figure 1A) as well as in tobacco leaves, as expected (Figure 1B, C). The labeled peroxisomes exhibited motility [see Additional Files 1 and 2] similar to that previously observed in *Arabidopsis *plants expressing the PST1 signal fused to GFP [5,25].

### YFP-class XI myosin-tail constructs label small plant cell organelles

To obtain myosin tail sequences, cDNA for myosin XI-5 (At MYA1), myosin XI-6 (At MYA2), myosin XI-8 (At XI-B), myosin XI-15 (At XI-I), myosin XI-16 (At XI-J) and myosin XI-17 (At XI-K) was obtained from *Arabidopsis thaliana *Columbia leaves by RT-PCR. Sequencing of two cDNAs for each gene confirmed that the gene model in Genbank was correct (data not shown). A few cDNAs exhibited minor nucleotide alterations, likely PCR errors, that did not affect the amino acid sequence. We did not detect any alternative splicing; however, more thorough studies using transcripts from a variety of tissues will be needed to determine whether these genes' transcripts exist in multiple forms.

**Figure 2:**
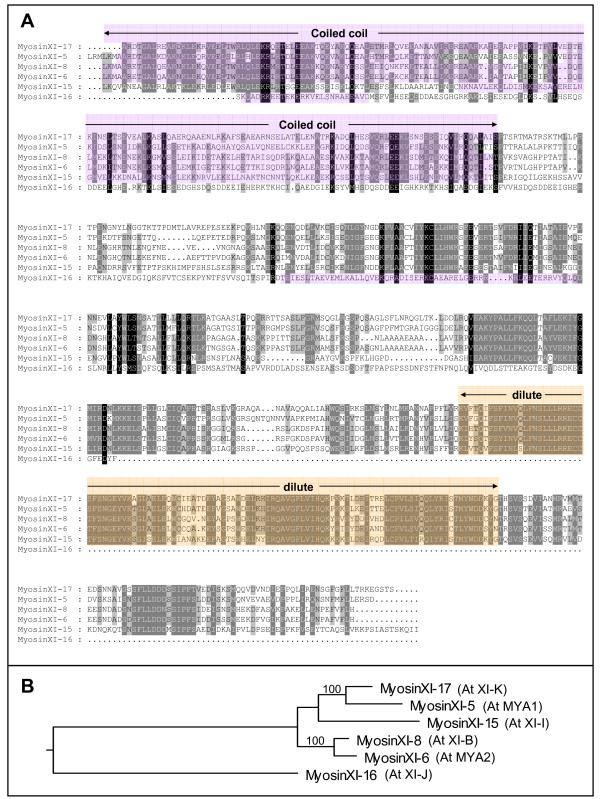
**Protein sequence similarity of myosin tails**. **A) **Protein sequence alignment of the six myosin tail constructs used in this study. Myosin XI-16 has the shortest tail. Identical and similar amino acids are highlighted in grayscale. Coiled-coil regions are marked in pink, and the dilute domains in yellow. Myosin gene numbers were derived from table 1 in Reddy and Day [15]. **B) **Cladogram based on similarity. Note the pairs myosinXI-17/XI-5 and myosinXI-6/XI-8. Myosin XI-15 is more similar to the myosinXI-17/XI-5 pair than to the other myosins. MyosinXI-16 is the most diverged. "100" refers to the confidence level of the analysis.

Amino acid alignment of the predicted tail sequence from the six cloned myosin tails shows a number of regions with high sequence identity (Figure 2A). Five of the 6 myosins exhibit significant sequence identity in the so-called "dilute" domain. The tail of myosin XI-16 is the shortest and lacks the dilute domain entirely (Figure 2A). On a phylogenetic tree based on full length *Arabidopsis *myosin alignments, Reddy and Day showed myosin XI-5 (At MYA1) and myosin XI-17 (At XI-K) are closely related and myosin XI-6 (At MYA2) and myosin XI-8 (At XI-B) also group together, while myosin XI-16 (At XI-J) and myosin XI-15 (At XI-I) are more diverged [15]. When we compare the amino acid sequences of the tail regions of the 6 myosins we have investigated, we note that the same two myosins within each of two pairs exhibit the highest similarity to one another (Figure 2B). Myosin XI-15 is more similar to the myosinXI-17/XI-5 pair than to the other myosins (Figure 2B).

**Figure 3:**
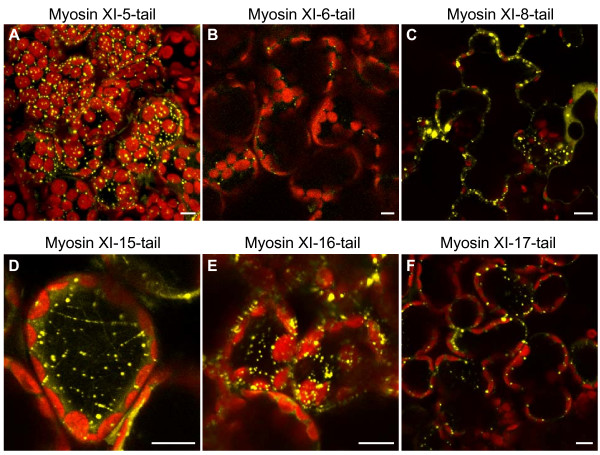
**Transient expression of YFP-class XI-tail myosins in tobacco leaves**. YFP-class XI-tail myosins were agroinfiltrated into tobacco leaves and the expression was observed 24 h later. **A) **Maximum projection (cell depth = 25 μm) of a z-stack series. **B-F) **Single confocal pictures. The pinhole was more opened in figures D and E. Yellow represents the YFP signal, chloroplasts are pseudo-colored in red. Bar = 10 μm.

We designed YPF fusion constructs in *Agrobacterium *vector pEarlygate 104 for each of the six myosin tails. The myosin tail constructs were designed in such a way that the N-terminal actin binding motor domain and the neck domain containing the IQ motif would be missing and replaced by the fluorescent protein. In melanocytes, comparable EGFP-tail fusions result in specific labeling of melanosomes [26]. The tail region in our constructs starts after the IQ motif and contains a coiled-coil region as well as a "dilute" domain near the C-terminus. The constructs were either transiently expressed in onion cells or in *A. thaliana *leaves by bombardment, or in tobacco leaves by agroinfiltration. 24 h after bombardment or 48 h after *Agrobacterium *infiltration, a yellow fluorescent signal was observed in the cells of the transformed tissues (Figure 3). The fluorescent signal was present in small vesicular structures. While only a few such vesicles are evident in some of the images of a single plane of the cell, they are quite numerous in most cells, as can be seen in a full projection view of several confocal z-stack sections from myosin XI-5-tail expressing tobacco leaf cells (Figure 3A). The fluorescent vesicles vary in size, with measurements revealing that they are between 0.5 – 3 μm in diameter. Thus their size is in the same range as the size of Golgi, mitochondria and peroxisomes, but smaller than chloroplasts and the non-green plastids in onion epidermal cells.

### Motility of compartments labeled with YFP-tail fusions

The labeled vesicles were observed to be motile in most cells examined [see Additional files 3, 4, 5, 6]. In other systems, expression of defective myosins lacking a functional motor domain results in cessation of movement of the normal cargo carried by the particular myosin, a dominant negative phenotype [27]. Evidently the abnormal YFP-tail myosin is not able to affect the function of a motor that can operate on the fluorescent organelle. If the fusion protein is unable to dimerize with an endogenous myosin and thereby destroy its function, then motility would be expected. Perhaps, unexpectedly, the fusion protein cannot dimerize with its homologous wild-type myosin through the coiled-coil domain but is still able to bind a cargo. The number of molecules of YFP-myosin present through transient expression may not be high enough to dimerize with most wild-type myosins to create a dominant-negative effect. Alternatively, the YFP-truncated myosin may be interacting with subcellular structures to which the wild-type myosin does not normally adhere. Another explanation is that that more than one class XI myosin is responsible for moving the same cargo. A further possible reason for the continued movement of the unidentified vesicles is that other motors move the same vesicle on microtubules; use of both the microfilaments and microtubules for motility has been reported for both Golgi stacks [28] and chloroplasts [9]. In animals, melanosomes are moved by both microtubule and actin motors [29]. However, the mobility of plant peroxisomes is prevented by actin and myosin inhibitors [5,6], so the continued movement of these organelles cannot be explained by use of microtubule motors.

**Figure 4:**
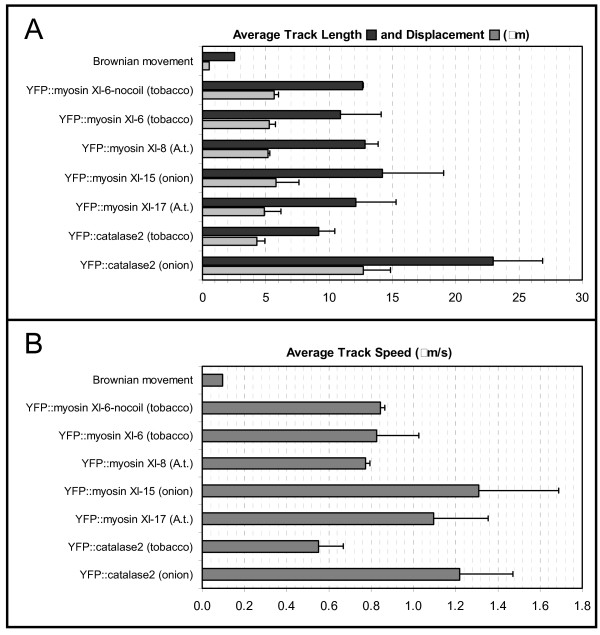
**Organelle Motility**. **A**) Track length, track displacement and (**B**) average speed of labeled organelles were analyzed on 2D time series. The data represents the mean values from at least 3 different experiments and a range of 300–3000 organelles were analyzed by construct. The plant material used for the labeled organelle motility determination is marked in brackets.

The average speed of the mobile vesicular structures and the length of the average track on which the organelles move differs depending on cell type and particular YFP-myosin expressed (Figure 4). The onion epidermal cells are larger and more elongate in shape that most mesophyll cells, and it is evident that peroxisomes exhibit a greater speed and track length in the non-green epidermal vs. green leaf cells. The difference in average organelle track speed in mesophyll cells exhibited by structures labeled by YFP-myosin XI-6 vs. XI-15 and XI-17 suggests that the populations of organelles labeled by these three myosins are not identical. It is clear that all these populations exhibit higher mobility and track length than vesicular structures exhibiting Brownian movement (Figure 4).

### Determining class XI myosin domains and tail lengths sufficient for targeting

**Figure 5:**
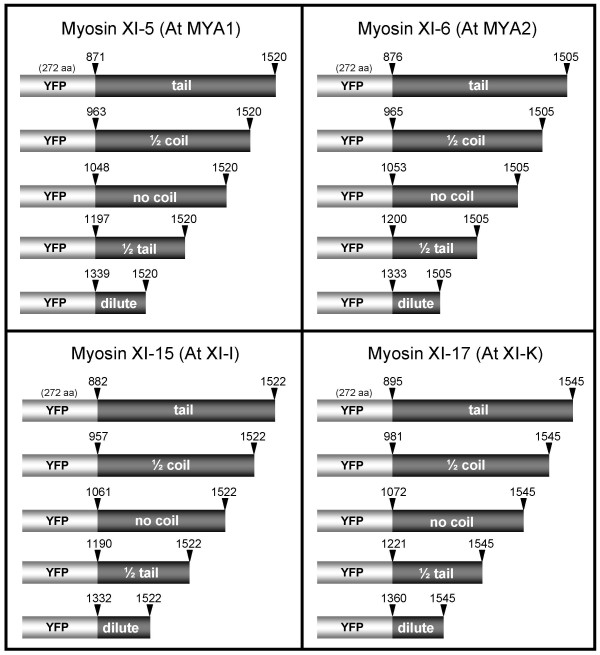
**Schematic representation of myosin constructs varying by tail length**. Schematic representation of the fusion constructs with different tail lengths for myosin XI-5, myosin XI-6, myosin XI-15 and myosin XI-17. The position of the amino acid based on the full length protein sequence is shown for each construct.

In order to determine which myosin tail length would be sufficient for labeling of discrete structures, we designed four different constructs varying by their length (Figure 5). The constructs start either 1) at the half coiled-coil region, or 2) just after the coiled-coil region, or 3) at the half length of the tail, or 4) just before the "dilute" domain. The C-terminus was kept intact in the deletion constructs because studies have shown that mouse MyoVa lacking the C-terminus was unable to co-localize with melanosomes in melanocytes [21]. The amino-acid position of each domain was obtained by searching the Pfam database [30]. Deletion constructs were made for Myosin XI-5 (At MYA1), Myosin XI-6 (At MYA2), Myosin XI-15 (At XI-I) and Myosin XI-17 (At XI-K).

**Figure 6:**
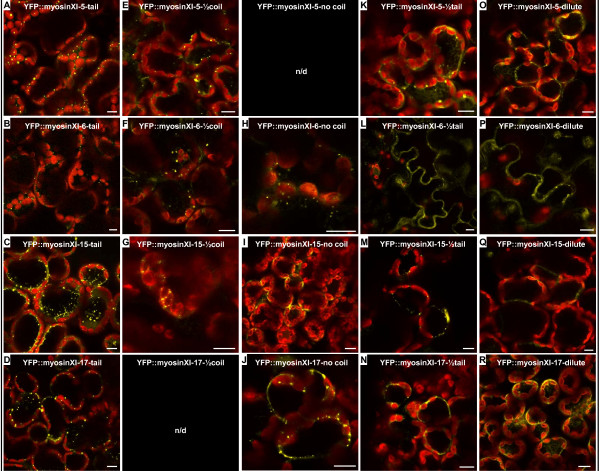
**Short myosin XI tail length fusion constructs are insufficient to be targeted to organelles**. Tobacco leaf cells after *Agrobacterium *infiltration with different myosin tail length fusion constructs. **A-D) **Complete tail constructs. **E-G) **1/2 coil constructs. **H-J) **Nocoil constructs. **K-N) **1/2 tail constructs. **O-R) **Dilute constructs. The yellow signal is from the YFP fusion constructs, red is chlorophyll autofluorescence. The shorter the tail, the more unspecific cytoplasmic labeling is observed. Rather few punctuate structures were observed in cells with 1/2 tail or dilute constructs. n/d = not determined. Bar = 10 μm.

After agroinfiltration, no major organelle targeting was obtained with any YFP-dilute construct (Figure 6O–R) as compared to full-length tail constructs (Figure 6A–D). The fluorescent signal with the globular dilute domain constructs was primarily in the cytoplasm. Proteins expressed from C half-tail fusion constructs also were usually not specifically targeted to organelles (Figure 6K–N). Sometimes a few punctate structures were labeled (Figure 6K, 6M), but the labeled entities did not move as freely as those observed after labeling with the complete tail constructs (data not shown). Myosin XI-6 and XI-17 YFP fusions without the coiled-coil domain were usually able to label vesicles (Figure 6H–J), but myosin XI-15 (At XI-I) without the coiled-coil region resulted in unspecific cytoplasmic labeling (Figure 6I). While myosin XI-5 and myosin XI-6 YFP fusions lacking half the coiled-coil region still labeled vesicles (Figure 6B, 6C), myosin XI-15 YFP 1/2-tail fusions resulted in only labeling of vesicles in very few cells (Figure 6G). For myosin XI-15, evidently the complete coiled-coil domain is necessary for targeting to a cargo. As observed with the full-length YFP-tail fusions, movement of the organelles occurred when they were labeled with half-coil and no-coil constructs [see Additional file 7].

### Comparison of the labeled compartments to Golgi, mitochondria, and peroxisomes

**Figure 7:**
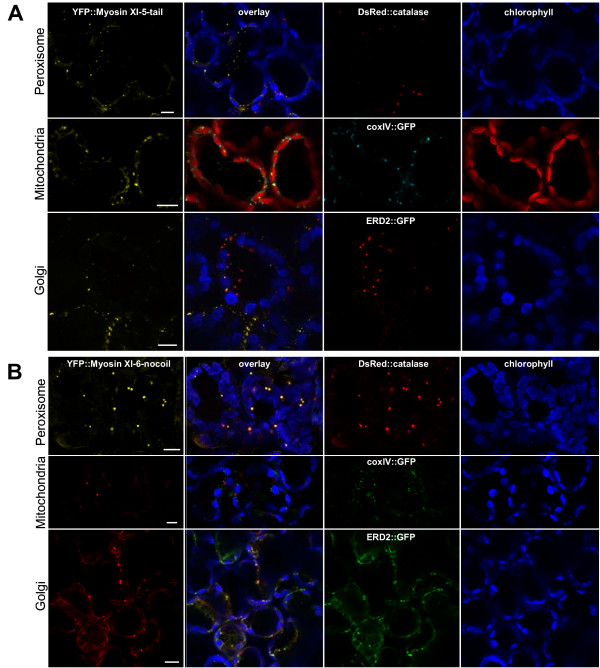
**Co-localization experiment with YFP::Myosin XI-5-tail and YFP::Myosin XI-6-nocoil against peroxisome, mitochondrial and Golgi markers**. Transient expression of **(A) ***YFP::Myosin XI-5-tail *or **(B) ***YFP::Myosin XI-6-nocoil *in tobacco leaves 48 h after *Agrobacterium *co-infiltration with either peroxisome marker *DsRed2::catalase*, or mitochondrial marker *coxIV::GFP*, or Golgi marker *ERD2::GFP*. All signals are pseudo-colored. Bar = 10 μm.

**Figure 8:**
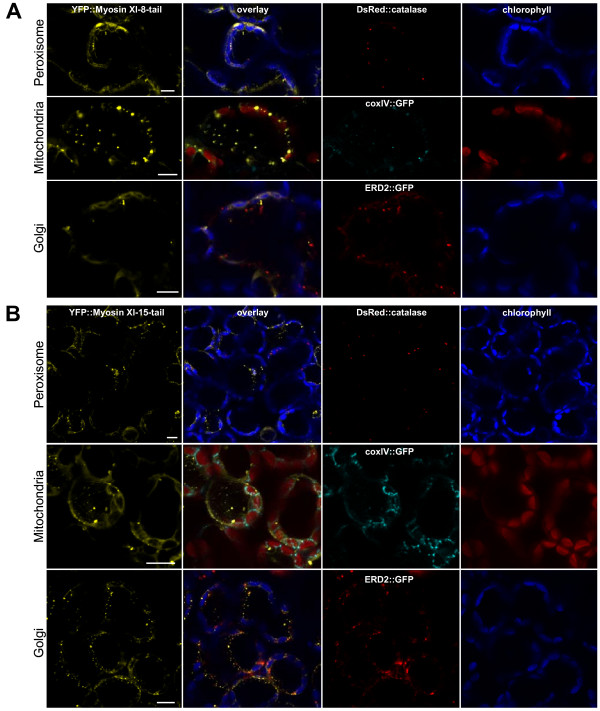
**Co-localization experiment with YFP::Myosin XI-8-tail and YFP::Myosin XI-15-tail against peroxisome, mitochondrial and Golgi markers**. Transient expression of **(A) ***YFP::Myosin XI-8-tail *or **(B) ***YFP::Myosin XI-15-tail *in tobacco leaves 48 h after *Agrobacterium *co-infiltration with either peroxisome marker *DsRed2::catalase*, or mitochondrial marker *coxIV::GFP*, or Golgi marker *ERD2::GFP*. All signals are pseudo-colored. Bar = 10 μm.

**Figure 9:**
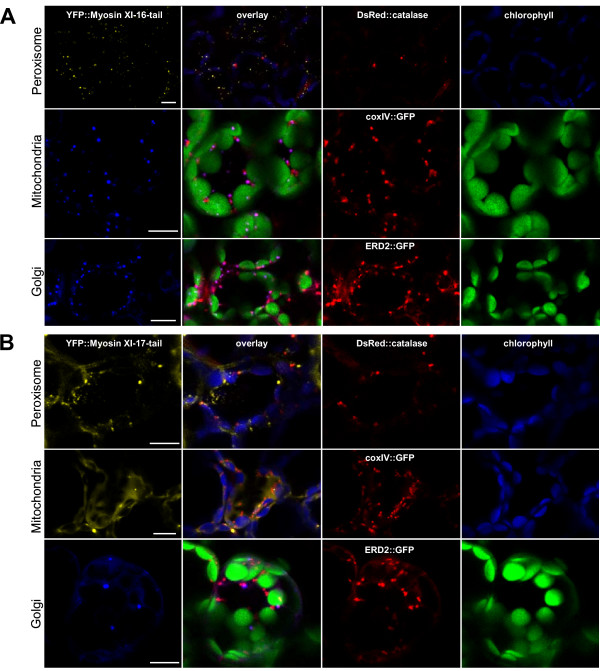
**Co-localization experiment with YFP::Myosin XI-16-tail and YFP::Myosin XI-17-tail against peroxisome, mitochondrial and Golgi markers**. Transient expression of **(A) ***YFP::Myosin XI-16-tail *or **(B) ***YFP::Myosin XI-17-tail *in tobacco leaves 48 h after *Agrobacterium *co-infiltration with either peroxisome marker *DsRed2::catalase*, or mitochondrial marker *coxIV::GFP*, or Golgi marker *ERD2::GFP*. All signals are pseudo-colored. Bar = 10 μm.

While the shape and size of the labeled structures following transient YFP-tail expression eliminated chloroplasts and non-green plastids from further consideration, the labeled structures were in the size range of Golgi bodies, mitochondria or peroxisomes. We transiently co-expressed fluorescent proteins known to label these compartments in order to determine whether any myosin XI-labeled compartments corresponded to these 3 organelle types. We expressed either the *catalase::DsRed2 *construct for peroxisomes, *coxIV::GFP *[2] for mitochondria, and *ERD2::GFP *[22] for Golgi labeling. Figures 7, 8, 9 show the result of the co-localization experiments for the six studied myosins.

For Myosin XI-6, the "nocoil" construct was expressed rather than the full tail region, because for unknown reasons, the shorter construct produced more consistent labeling. YFP::Myosin XI-6-nocoil (Figure 7B) co-localized with the peroxisomal catalase::DsRed2 marker but not with the mitochondrial or Golgi body marker. The YFP::Myosin XI-5-tail (Figure 7A), YFP::Myosin XI-8-tail (Figure 8A) and YFP::Myosin XI-15-tail (Figure 8B) do not co-localize with any of the markers. YFP::Myosin XI-16-tail did not co-localize with the peroxisome marker, but slight overlaps were observed with the mitochondrial and Golgi markers (Figure 9A). We did not detect any conclusive co-localization with any marker for YFP::Myosin XI-17-tail (Figure 9B), though occasionally there were a few slight overlaps with Golgi stacks.

The co-localization of YPF::myosin XI-6-nocoil confirms the finding that this myosin interacts with peroxisomes, as previously reported in studies using anti-MYA2 (XI-6) antibodies in transgenic *Arabidopsis *plants expressing a GFP-tagged peroxisomal targeting signal peptide [31]. None of the other five myosin constructs tested co-localized with this peroxisome marker. The overlap of the signal of YFP::myosin XI-16-tail with the Golgi and mitochondrial markers is suggestive but not entirely conclusive, because YFP and GFP are difficult to separate due to overlapping excitation peaks. Further studies with additional control fluorescent protein fusions need to be undertaken in order to verify whether YFP::myosin XI-16 interacts with Golgi bodies and mitochondria. Nevertheless, our experiments do show that motile vesicles are reproducibly labeled with myosin YFP-tail fusions. The identity of these vesicles can be further probed in the future through co-localization with proteins and dyes known to label various compartments involved in vesicular transport processes.

### Occasional labeling of linear structures

**Figure 10:**
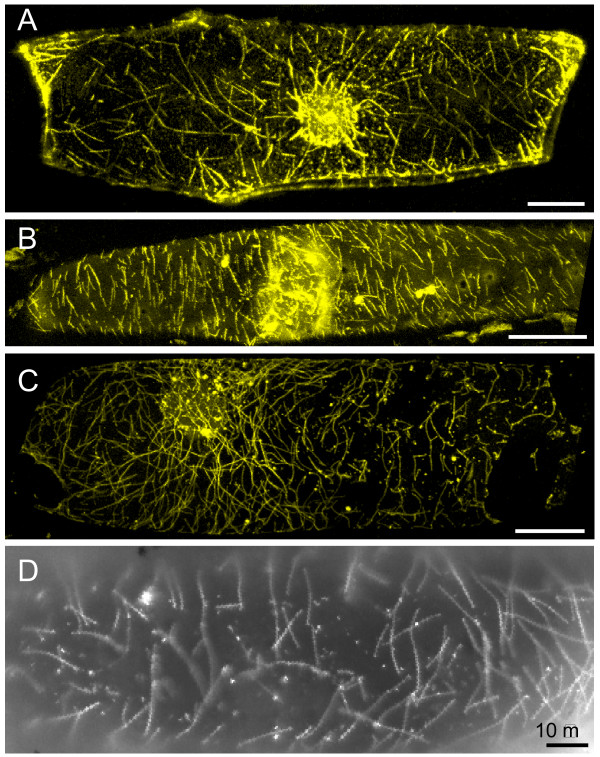
**Appearance of filamentous structures labeled by myosin constructs**. **A) **Maximum projection (z = 121 μm) of serial confocal sections of an onion epidermal cell transiently expressing *YFP::myosin XI-5- 1/2 coil*. **B) **Maximum projection (z = 18 μm) of serial confocal sections of an onion epidermal cell transiently expressing *YFP::myosin XI-17-tail*. **C) **Maximum projection (z = 24 μm) of serial confocal sections of an onion epidermal cell transiently expressing *YFP::myosin XI-15-tail*. Bar in A-C = 50 μm. **D) **Close-up of the microtubule-like structures in an onion epidermal cell transiently expressing *YFP::myosin XI-15-tail*.

Instead of the reproducible organelle labeling seen in most experiments, on rare occasions we observed long filamentous structures with the characteristic appearance of actin filaments (Figure 10A, 10C). The linear structures were similar to actin microfilaments found in GFP-FABD2 expressing *Arabidopsis *seedlings [32] or GFP-talin cells [33]. Such labeling was unexpected because the actin-binding motor domain is missing in the fusion proteins. However, if heterodimerization between the YFP::myosin-tail fusions and endogenous myosins sometimes occurs, then perhaps the myosin heterodimer could interact with actin filaments, resulting in labeling. Perhaps the rare cells giving filamentous structures were expressing unusually high amounts of the YFP-tail fusion proteins. Because the proteins are being expressed transiently from T-DNA following agroinfilitration, different cells are likely to accumulate different amounts of the YFP-myosin proteins.

Not all linear structures appeared similar to microfilaments. Sometimes we observed short linear structures (Figure 10B, 10D) that appeared more similar to microtubules than microfilaments [34]. Usually a myosin would not be expected to react with microtubules. However, there is evidence that motors sometimes link the actin and tubulin cytoskeleton. The *Drosophila *myosin VI interacts with a microtubule plus-end-binding protein [35,36]. Even though peroxisomes move on microfilaments [37], the plant peroxisomal multifunctional protein was shown to bind cortical microtubules *in vitro*, and peroxisomes and microtubules were observed to interact [38]. In CHO cells, peroxisome association with microtubules was also described [39]. Thus, for unknown reasons, perhaps the myosin XI YFP-tail constructs can sometimes interact with microtubules.

## Conclusion

Six different fluorescent myosin XI-tail fusion proteins can label small vesicular structures. Of the 6 fusion proteins analyzed, only YFP::myosin XI-6 evidently interacts with peroxisomes. Most of the C-terminus past the motor domain and IQ repeats was necessary for the myosin to be targeted to an organelle. N-terminal deletions past the coiled-coil region results resulted in loss of specific labeling of vesicles. Unlike in animal systems, the defective myosins did not disrupt motility of the labeled organelles. Either the YFP-myosins can bind to organelles not usually mobilized by the corresponding normal myosins, were not present in adequate concentrate to saturate the normal myosin subunits, or there is redundancy in the motor machinery that is responsible for intracellular trafficking.

## Methods

### RNA extraction, RT-PCR, plasmid construction

*Arabidopsis thaliana *cv Columbia leaf RNA extraction was performed using RNeasy Plant Mini Kit (Quiagen) followed by reverse transcription using Omniscript reverse transcriptase (Qiagen) with an oligo-dT_17 _primer. Myosin XI tails were amplified by PCR using the Taq PCR Master Mix Kit (Quiagen) with the following primer pairs:

For Myosin XI-5 (At MYA1; AT1g17580): Myo5fwd 5'GCTTAGAATGCTGAAAATGGCTGC3' and Myo5rev 5'GGATCTGACCTTTCCAACAAGAAC3';

For Myosin XI-6 (At MYA2; AT5g43900): Myo6fwd 5'GCTTAAGATGGCTGCTAGAG3'and Myo6rev 5'AGTGCAAGAATACAAATGCTGG3';

For Myosin XI-8 (At XIB; AT1g04160): Myo8fwd 5'CTTAAGATGGCTGCTCGAG3'and Myo8rev 5'AGTGCAAGAATACGAATTC3';

For Myosin XI-15 (At XI-I; AT4g33200): Myo15fwd 5'CTTAAACAGGTTGCTAATGAAGC3' and Myo15rev 5'TCAAATGATCTGCTTTGAGGTTG3';

For Myosin XI-16 (At XIJ; AT3g58160): Myo16fwd 5'TCAAAGCAGGCTGACAGAA3' and Myo16rev 5'TCAAAAGTAATCTTCGAAGCCC3';

For Myosin XI-17 (At XIK; AT5g20490): Myo17fwd 5'CGCACGAGACACAGGAGCCCTTA3' and Myo17 rev 5'GGCGATGTACTGCCTTCTTTACG3'.

PCR products were cloned into a pCR2.1-TOPO T/A vector (Invitogen) and sequenced. Cloned myosin tails and cropped tail constructs were further PCR amplified with primers (Table 1) allowing GATEWAY directional cloning using AccuPrime™ *Pfx *DNA Polymerase (Invitogen). PCR products were cloned into a pENTR/D TOPO vector (Invitrogen), and LR clonase reactions between the entry vectors and the destination vector pEarleyGate104 [40] were performed according to manufacturer's instructions (Invitrogen). pENTR/D vectors containing the genes of interest were digested with *Mlu*I prior the LR reaction.

**Table 1:** Primers for GATEWAY directional cloning

**Primers**	**Myosin XI-5 (At MYA1)**	**Myosin XI-17 (At XI-K)**
tail	CACCCTTAGAATGCTGAAAATGGCTG	CACCGCACGAGACACAGGAGC
1/2 coil	CACCGTTGTTGTTGAAGATACAG	CACCGTATTGGTTGAGGATACTG
no coil	CACCACTCTTACCATCTCACCGAC	CACCACCAGCAGAACTATGG
1/2 tail	CACCAGACGTCGGGGAATGCCTTC	CACCCAGAGGCGAAGAACAACATCAGC
dilute	CACCGTGTTCGGGCAGATAT	CACCGTATTCACACAAATATTCTCC
reverse	TCAATCTGACCTTTCCAACAAG	TTACGATGTACTGCCTTCTTTACG

	**Myosin XI-6 (At MYA2)**	**Myosin XI-15 (At XI-I)**

tail	CACCCTTAAGATGGCTGCTAGAG	CACCCTTAAACAGGTTGCTAATG
1/2 coil	CACCGTGGTTGATCAGGAATT	CACCGCGGTACTTGAAAAGC
no coil	CACCGTGAGGACAAATCTAGGAC	CACCTCCCCAGAGAGAATAGGAC
1/2 tail	CACCGCTTCAACTTCTTTATTTGG	CACCGCTTCAGCACTTCTATGC
dilute	CACCACTTTCTCATACATTAACG	CACCCTTGTGACTCAGGTTTTCTC
reverse	CTAGTGCAAGAATACAAATGCTGG	TCAAATGATCTGCTTTGAGGTTG

	**Myosin XI-8 (At XI-B)**	**Myosin XI-16 (At XI-J)**

tail	CACCCTTAAGATGGCTGCTCG	CACCTCAAAGCAGGCTGACAG
reverse	CTAGTGCAAGAATACGAATTCTGG	TCAAAAGTAATCTTCGAAGCCC

All primers are listed 5'→3'. Forward primers contain the four bases CACC allowing directional cloning into pENTR/D TOPO vector (Invitrogen).

At Catalase 2 cDNA (At4g35090) was obtained by RT-PCR using the following primer pairs: 5'ATGGATCCTTACAAGTATCGTC3' and 5'CTAGATGCTTGGCCTCACG3'. The PCR product was cloned into a pCR2.1-TOPO T/A vector (Invitogen), and sequenced. The gene was then amplified with the following primers for allowing a directional cloning into the pENTR/D TOPO vector (Invitrogen): 5'CACCGATCCTTACAAGTATCGTC3' and 5'TTAGATGCTTGGTCTCACG3'. The gene was fused to YFP in the pEarleyGate104 [40] by LR reaction (Invitrogen). The catalase cDNA was also fused downstream of DsRed2 in the binary pGDR vector [41] by using the *Kpn*I restriction site. Primers containing *Kpn*I restriction sites (bold) were used: 5'GG**GGTACC**ATGGATCCTTACA3' and 5'GG**GGTACC**TAGATGCTTGGTC3'. The purified PCR product was digested by *Kpn*I and cloned into the *Kpn*I linearized and dephosphorylated pGDR vector using T4 ligase (Invitrogen). Positive clones were screened by digestion profile and confirmed by sequencing.

### Sequence alignment and cladogram for myosin tails

Myosin tail sequences were aligned by using MultAlign [42], and edited with GeneDoc [43]. Coiled-coil regions were predicted with COILS [44] in the SMART module [45,46] and "dilute" domains were identified with Pfam [30]. Amino acid similarity tree (cladogram) was made in Megalign (DNAStar) with the Clustal W method.

### Transient expression

Onion cells or *Arabidopsis thaliana *leaves were bombarded with plasmid-coated tungsten particles using a Model PDS 1000/He Biolistic Particle Delivery System™ (BioRad, Hercules, CA, USA) according to manufacturer's instructions. Plasmid DNA (2 μg for each shot) was precipitated on tungsten particles, and onion or *A. thaliana *epidermal cells were bombarded twice. The expression was observed 24 h after bombardment.

*Agrobacterium*-mediated transient expression was performed as previously described [47]. Briefly, the binary GATEWAY-plasmid vectors were transformed by electroporation into *A. tumefaciens *strain C58C1 carrying the virulence helper plasmid pCH32 [48]. The transformants were inoculated into 5 ml LB medium supplemented with 50 μg ml^-1 ^kanamycin and 5 μg ml^-1 ^tetracycline and grown at 28°C overnight. Cells were precipitated and resuspended to an OD of 0.5 in solution containing 10 mm MgCl_2_, 10 mm MES pH 5.6 and 150 μm acetosyringone. The cells were left at room temperature on the bench for 2 h before infiltration into *N. bentamiana *leaves. Observation was done 48 h after infiltration.

### Confocal laser scanning microscopy

Confocal microscopy was performed on a Leica DMRE-7 (SDK) microscope equipped with a TCS-SP2 confocal scanning head (Leica Microsystems Inc., Bannockburn, IL, USA). Images were collected with a Leica 63 × HCX PL APO water immersion objective (NA = 1.20). YFP was excited at 514 nm with an ArKr laser, and emission was detected between 525 and 625 nm. Chlorophyll was excited at 488 nm with an ArKr laser and emission was detected between 640 and 715 nm. DsRed was excited at 514 with an ArKr laser or at 543 nm with a GreNe laser and emission was detected between 563 and 660 nm. GFP was excited at 488 with an ArKr laser and emission was detected between 498 and 515 nm. For co-localization experiments, images were sequentially collected in order to minimize cross-talk of GFP and YFP. For time lapse series YFP and chlorophyll were both excited at 488 nm and pictures were collected without frame averages. Organelle motility was measured with the ImarisTrack module from the Image Analyzing Software Imaris 5.0 (Bitplane AG, Zurich, Switzerland). Labeled organelles were detected with the spot function and tracks were analyzed by using the autoregressive motion algorithm. Brownian movement was tracked with the Brownian motion algorithm.

## Authors' contributions

DR performed the cloning, transgene expression, microscopic observations and analysis and drafted the manuscript. MRH conceived of the study, participated in its design and coordination, and edited the manuscript. Both authors read and approved the final manuscript.

## Supplementary Material

Additional file 1 **Peroxisome movement in onion cells visualized with a YFP::catalase2 construct**. Onion cells were transiently transformed by bombardment with *YFP::catalase2 *DNA, and expression was observed 24 h later on a confocal microscope. Time series were collected and the movie displays the pictures with a 20-fold acceleration. Click here for file

Additional file 2 **Peroxisome movement in tobacco leave cells visualized with a YFP::catalase2 construct**. *N. benthamiana *leaves were infiltrated with *Agrobacterium *carrying a *YFP::catalase2 *construct. Expression was observed 48 h later on a confocal microscope. Time series were collected and the movie displays the pictures with a 16-fold acceleration. Click here for file

Additional file 3 **Movement of YFP::myosin XI-6-tail labeled organelles**. *N. benthamiana *leaves were infiltrated with *Agrobacterium *carrying a *YFP::myosin XI-6-tail *construct. Expression was observed 48 h later on a confocal microscope. Time series were collected and the movie displays the pictures with a 16-fold acceleration. Click here for file

Additional file 4 **Movement of YFP::myosin XI-8-tail labeled organelles**. *Arabidopsis thaliana *leaves were bombarded with *YFP::myosin XI-8-tail *DNA and expression was observed 24 h later on a confocal microscope. Time series were collected and the movie displays the pictures with a 16-fold acceleration. Click here for file

Additional file 5 **Movement of YFP::myosin XI-15-tail labeled organelles**. Onion cells were transiently transformed by bombardment with *YFP::myosin XI-15-tail *DNA, and expression was observed 24 h later on a confocal microscope. Time series were collected and the movie displays the pictures with a 10-fold acceleration. Click here for file

Additional file 6 **Movement of YFP::myosin XI-17-tail labeled organelles**. *Arabidopsis thaliana *leaves were bombarded with *YFP::myosin XI-17-tail *DNA and expression was observed 24 h later on a confocal microscope. Time series were collected and the movie displays the pictures with a 16-fold acceleration. Click here for file

Additional file 7 **Movement of YFP::myosin XI-6-nocoil labeled organelles**. *N. bentamiana *leaves were infiltrated with *Agrobacterium *carrying a *YFP::myosin XI-6-nocoil *construct. Expression was observed 48 h later on a confocal microscope. Time series were collected and the movie displays the pictures with an 8-fold acceleration. Click here for file
